# Mapping IS*6110* in high-copy number *Mycobacterium tuberculosis* strains shows specific insertion points in the Beijing genotype

**DOI:** 10.1186/1471-2164-14-422

**Published:** 2013-06-25

**Authors:** Henar Alonso, Sofía Samper, Carlos Martín, Isabel Otal

**Affiliations:** 1 Grupo de Genética de Micobacterias. Departamento de Microbiología, Medicina Preventiva y Salud Pública, Universidad de Zaragoza, C/ Domingo Miral sn. 50009, Zaragoza, Spain; 2 Laboratorio de Investigación Molecular, Hospital Universitario Miguel Servet, IIS Aragón, Zaragoza, Spain; 3 CIBER Enfermedades Respiratorias (CIBERES), Madrid, Spain; 4 Institut de Pharmacologie et de Biologie Structurale, UMR5089 CNRS, 205 Route de Narbonne, BP 64182, 31077 Toulouse France

**Keywords:** IS*6110*, Insertion points, *M. tuberculosis*, Beijing genotype

## Abstract

**Background:**

*Mycobacterium tuberculosis* Beijing strains are characterized by a large number of IS*6110* copies, suggesting the potential implication of this element in the virulence and capacity for rapid dissemination characteristic of this family. This work studies the insetion points of IS*6110* in high-copy clinical isolates specifically focusing on the Beijing genotype.

**Results:**

In the present work we mapped the insertion points of IS*6110* in all the Beijing strains available in the literature and in the DNA sequence databases. We generated a representative primer collection of the IS*6110* locations, which was used to analyse 61 high-copy clinical isolates. A total of 440 points of insertion were identified and analysis of their flanking regions determined the exact location, the direct repeats (DRs), the orientation and the distance to neighboring genes of each copy of IS*6110*. We identified specific points of insertion in Beijing strains that enabled us to obtain a dendrogram that groups the Beijing genotype.

**Conclusions:**

This work presents a detailed analysis of locations of IS*6110* in high-copy clinical isolates, showing points of insertion present with high frequency in the Beijing family and absent in other strains.

## Background

The insertion sequence (IS) *6110* is specific for the *Mycobacterium tuberculosis* complex (MTBC) [1]. *M*. *tuberculosis* strains typically contain multiple copies of IS*6110* (up to 25 per genome) [2], although strains with only a single copy or no copies have also been identified [3-5]. In contrast, *M*. *bovis* strains are characterized by having a low copy number, with *M*. *bovis* BCG substrains having either one or two copies [6]. The high variability in copy number and location of IS*6110*, as well as its stability over time, renders IS*6110* a useful diagnostic and epidemiological tool. Moreover, in some cases, the location of a copy that is specific for a strain can be used for the rapid identification and differentiation of that particular strain from other isolates [7].

Without a known insertion target, IS*6110* has been found within ORFs and intergenic regions [2,8]. The IS*6110* locations along the genome are not equally distributed, being found more often in some regions, while completely absent in others [9-11]. In single-copy strains of *M*. *tuberculosis* and *M*. *bovis*, IS*6110* is always present in the conserved 36-bp array, designated the Direct Repeat region (DR region) [12] and, with minor exceptions, all members of the MTBC carry a copy integrated in this locus. General hot-spots of IS*6110* include IS*1547* in *iplA*-*iplB* region [13,14], the phospholipase C region (locus *plcABC* and the *plcD* gene) [15,16], members of the PPE gene family [17] and the origin of replication (*oriC*) [18,19].

One of the most relevant and better studied mechanisms of genome change is IS-mediated. It is considered that about 5% to 15% of the spontaneous mutations in the bacterial genome are due to changes in IS locations [20]. Transposition is one of the more common mechanisms used by IS to move along genome, whereas recombination between two IS copies can lead to genome deletions [21]. Hence, an IS could either play a role as a potential enemy or a helpful ally affecting the fitness of the bacterium. IS*6110* insertions, genetic reorganizations and deletions are some of the mechanisms proposed to be responsible for differences in the virulence phenotypes among *M*. *tuberculosis* strains. IS*6110* has been associated with participation in adaptation to a particular host [22], activation of genes during infection [23], evolution [21] and in activation of downstream genes with an orientation-dependent activity promoter [10,23,24].

The Beijing strains are considered one of the most successful families in tuberculosis transmission. Three features that characterize the Beijing family are related to the IS*6110* insertion sequence. These include: i) the presence of a copy in the *ori*C known as insertion A1, ii) the deletion of the region of difference (RD) 207, and iii) a similar IS*6110*-RFLP multiband pattern profile [25]. Members of this genotype are characterized by a high-copy number of IS*6110*[26], suggesting the potential implication of this element in the special characteristics of this family related to virulence and capacity for rapid dissemination.

In the present work, we conducted an in-depth study of the points of insertion of IS*6110* in selected Beijing clinical isolates and in the Beijing strains available in the literature and in the DNA sequence databases. With the obtained locations, we generated a representative primer collection of Beijing-IS*6110* points of insertion, which was used to analyse 61 high-copy clinical isolates. We established the specific and shared copy locations in the studied strains and constructed a dendrogram allowing the accurate classification of Beijing genotype strains.

## Results and discussion

### Generation of a primer collection of IS*6110* insertion points

**Figure 1:**
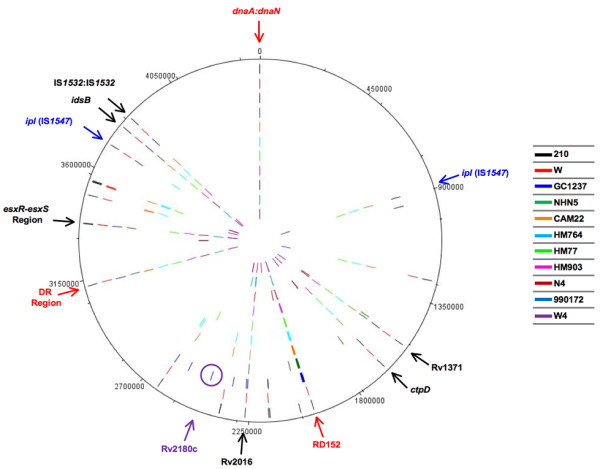
**Distribution of IS*6110 *insertion sequence throughout the genome of *M. tuberculosis *Beijing strains.** The located copies of IS*6110* in NHN5, HM77, HM903, HM764, 990172, W4, N4 and CAM22 Beijing strains were plotted in H37Rv genome and represented in different concentric circles. IS*6110* locations present either in all strains (red arrows) or with high frequency (black arrows) are indicated; *ipl* hot-spots are pointed with blue arrows. In this DNA plotter, the IS*6110* locations of GC1237 [10], W and 210 Beijing strains [31] were also represented as IS*6110* reference points of insertion. The unique insertion of IS*6110* of GC1237 [10] (within Rv2180c) is surrounded by a purple circle.

Firstly, we studied the points of insertion of IS*6110* in eight representative Beijing strains (NHN5, HM77, HM903, HM764, 990172, W4, N4 and CAM22) by LMPCR. Twenty-two new genomic insertion points were obtained by this technique. Primers for the identified new IS*6110* locations were designed and added to primers used in a previous work for localization of IS*6110* copies in the Beijing strain GC1237 [10]. Through LMPCR and PCR, a total of 106 (45 different) IS*6110* insertion points were obtained and plotted on the H37Rv genome map. The shared insertion points by these eight strains and the three reference Beijing strains (GC1237, 210 and W [10,31]) were obtained (Figure 1 and Additional file 1: Tables S4 and S5).

**Figure 2:**
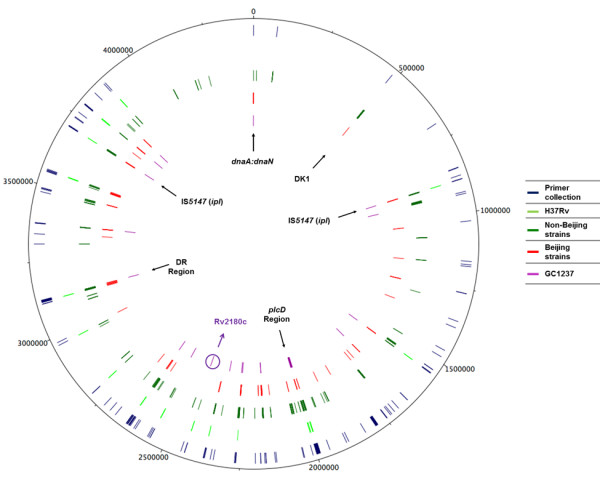
**Amplified regions with primer collection and distribution of points of insertion of IS*6110 *of *M.tuberculosis *sequenced genomes (GeneBank).** The first circle (blue) represents the distribution of the regions which can be amplified with the primer collection generated in this work. The second concentric circle (fluorescent green) represents the genomic locations of IS*6110* in the reference strain H37Rv. The third and fourth circles represent the distribution of this sequence in non-Beijing strains (green) and in Beijing strains (red) respectively and the fifth concentric circle (purple) correspond to the genomic points of insertion of IS*6110* in GC1237 used as another reference strain. All the points of insertion and amplified regions are plotted in *M*. *tuberculosis* H37Rv genome. The hot-spots: IS*1547*, *plcD* region, DR region, *dnaA*:*dnaN* region and DK1 region are indicated by arrows. The unique insertion of IS*6110* of GC1237 [10] (within Rv2180c) is surrounded by a purple circle.

In addition, we analysed the IS*6110* insertion points in 43 reference sequenced strains in the GeneBank comparing their sequenced fragments with H37Rv genome. We obtained a total of 486 points of insertion (Additional file 1: Table S3), which were plotted on the H37Rv genome map and were grouped in two concentric circles: one corresponding to non-Beijing strains (green circle) and another to Beijing strains (red circle) (Figure 2).

In addition to the GeneBank analysis, we reviewed the available literature [10,11,13,16-18,20,22-24,27-35] for new locations of IS*6110* in Beijing strains, for which new pairs of primers were designed. In addition, to detect whether the preferred insertion site of IS*6110*, DK1 region [28], in low-copy number strains (LCS) (< 7 copies) is present or absent in high-copy number strains (HCS), a specific pair of primers was designed. Moreover, the primers used to amplify locations of IS*6110* in *M*. *bovis* human isolates [22] were included, as the host in both cases is the same. With all these pairs of oligonucleotides we generated a primer collection (238 oligonucleotides) (Additional file 1: Table S2). Figure 2 depicts the amplified regions with the primer collection (first concentric blue circle) showing quite a homogeneous distribution along the *M*. *tuberculosis* genome.

### Locations of IS*6110* in Beijing genotype

All the Beijing strains (8 representative strains, GeneBank and literature) presented the three characteristic IS*6110* locations of Beijing family: the insertion A1, between Rv1754c-Rv1765c genes, which corresponds to deleted RD152, and in the DR region (Figure 1). Of note, two copies of IS*6110*, both in the same orientation, were detected in the DR region in CAM22 strain.

Interestingly, when we compared the eight representative strains, we observed six sites of insertion present with high frequency in the genes Rv1371, *ctpD* (Rv1469c), Rv2016 and *idsB* (Rv3383c), between the two IS*1532* and in *esxR*-*esxS* region. These 6 insertion points are also present in the three reference Beijing strains (GC1237, 210 y W) [10] (Figure 1 and Additional file 1: Table S5).

According to some authors, it is frequent to find at least one copy of IS*6110* in the NTF region [36] in strains belonging to Beijing family and some members of this family may have a second insertion within this locus such as the MDR W strain [37,38]. It has been proposed that the absence of IS*6110* in the NTF locus may be associated with ancestral Beijing genotype sublineages [39] and in the course of evolution some strains (“modern” sublineages) have acquired the insertion of IS*6110* in this region [37]. The eight analyzed strains do not present IS*6110* in this region, which according to some authors, would be classified as ancestral.

### Distribution of IS*6110* among Beijing and non-Beijing strains

We studied the distribution of IS*6110* in the eight analysed Beijing strains and in the sequenced *M*. *tuberculosis* genomes (from the GeneBank and in the literature) and observed that the locations were random (Figure 1 and Figure 2). However, the presence of numerous preferential integration loci of IS*6110* were also detected (some examples are indicated by arrows in Figure 2) in Beijing and non-Beijing strains, corroborating favored integration regions common among *M*. *tuberculosis* strains. In all strains, this element was found within the DR region. In the non-Beijing strain SUMu003, a copy was observed in a different point other than A1 in the *dnaA*:*dnaN* region, indicating that the presence of an IS*6110* in this region is not exclusive to the Beijing family (Figure 2). This result is in agreement with studies which show different locations of IS*6110* in the *dnaA*:*dnaN* region in non-Beijing strains [18,19,40]. The amplification of this entire intergenic region is a useful tool to detect Beijing isolates, but another genomic feature of this genotype is also necessary to avoid potential false positive results.

IS*6110* was found inserted more often in some genomic regions (e.g., 1800000 bp - 2700000 bp) than in others that could be more abundant in essential genes (e.g., the region near *oriC*) (Figure 2). In fact, if an IS*6110* transposes in essential regions, the outcome of this event would not be observed. These findings are in agreement with previous studies of chromosomal distribution of IS*6110*[11,27,30,32].

### Mapping IS*6110* in 61 HCS

With the aim of studying the IS*6110* insertion points and compare them between Beijing and non-Beijing HCS strains, we analysed 61 *M*. *tuberculosis* clinical isolates selected for their high copy number. The isolates comprised 44 non-Bejing and 17 Beijing strains, including the eight previously studied in this work.

**Figure 3:**
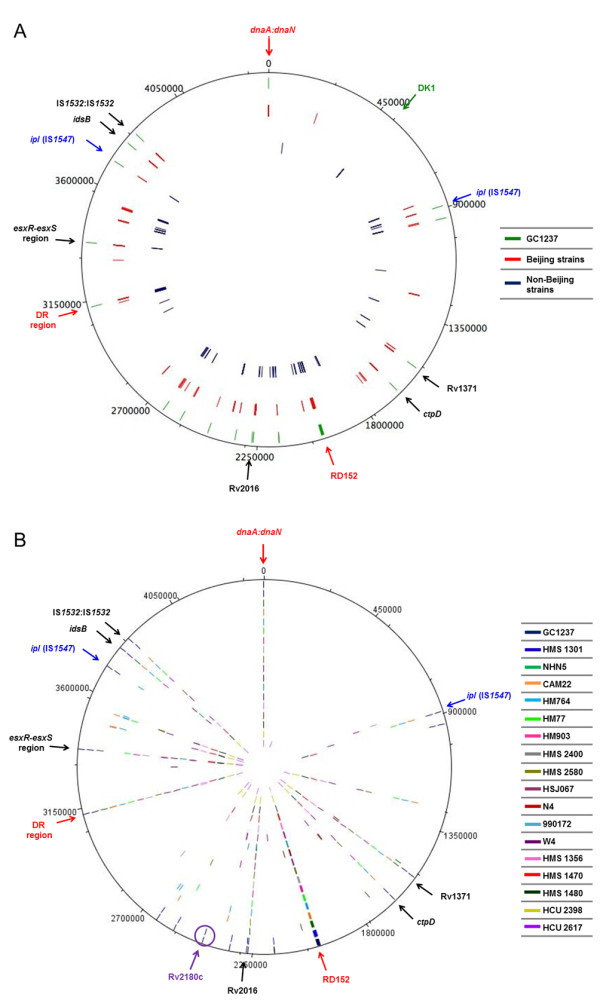
**Distribution of IS*6110 *thorough the *M. tuberculosis *genome of the studied strains.** (**A**). The obtained locations of IS*6110* of each strain were plotted in *M*. *tuberculosis* H37Rv genome and represented in three concentric circles: the blue one corresponds to the IS*6110* locations in non-Beijing strains. The red one represents the IS-locations in Beijing strains and the green one corresponds to the locations of IS*6110* in GC1237 strain. The general hot-spots of *M*. *tuberculosis* are indicated by blue/red arrows, the DK1 region is indicated by green arrow and possible specific hot-spots of Beijing genotype are indicated with black arrows. (**B**) The IS*6110* locations obtained in the 17 Beijing strains were plotted in *M*. *tuberculosis* H37Rv genome and represented each one in a concentric circle. The IS*6110* locations of GC1237 were included as control. The general hot-spots are indicated by blue/red arrows and with black arrows the IS*6110*-Beijing-hot-spots.

The study was carried out by PCR using the entire primer collection (138 reactions for each clinical isolate) (Additional file 1: Table S2) and when the amplified fragment indicated that the IS*6110* was present, the product was sequenced with IS61 and IS62 primers (Additional file 1: Table S2). By this method we obtained the two flanking regions of all the copies. By analysis of the sequences, we obtained the insertion site, the flanking 3–4 bp direct repeats (DRs), the orientation and the distance to neighboring genes of each copy of IS*6110* (Additional file 1: Tables S4 and S5). A total of 440 (160 different) insertion points were obtained (Additional file 1: Tables S4 and S5) and plotted on the genomic map of H37Rv and represented with DNA plotter in two different circles, red (insertions in Beijing strains) and blue (non-Beijing strains) (Figure 3A). The locations of IS*6110* in the reference Beijing strain GC1237 were included in a separated circle (green) as reference points of insertion.

From the 61 analysed clinical isolates, we localized a lower number of insertion points in non-Beijing strains (1 to 4) than in Beijing strains (at least 10). This finding is probably because the primer collection is based mainly in points of IS*6110* from Beijing strains. All strains presented one copy of IS*6110* in the DR region. Of the non-Beijing strains, 90.9% presented the same insertion site in this region. The remaining non-Beijing strains presented a copy in different points within this region (Additional file 1: Table S4), which is in agreement with other authors who have observed 16 different locations within the DR region [11].

Other special locations that we studied in HCS were the DK1 insertion point and the sites of IS*6110* in *M*. *bovis* clinical isolates [22]. According to Fomukong *et al*. [28], the DK1 (*mmpS1* gene) insertion point is highly preferred in LCS and the authors defend the idea that its prevalence decreases in HCS, suggesting a separate lineage for HCS and LCS [28,29]. No IS*6110* was detected in the DK1 locus in all 17 Beijing strains analysed (Figure 3A). Six of the 44 studied non-Beijing strains presented an IS*6110* in the exact DK1 site (Additional file 1: Table S4) (Figure 3B). The high-copy number in these strains could be the result of transposition of another IS*6110*. Additionally, we found that none of the studied *M*. *tuberculosis* isolates have an equal insertion point of IS*6110* as the *M*. *bovis* clinical isolates, supporting that *M*. *bovis* and *M*. *tuberculosis* evolved separately from a common precursor at an early stage.

The insertion of IS*6110* in the characteristic Beijing spot RD152 was also observed in non-Beijing strains (region between Rv1754c-Rv1765c), indicating that this region is not exclusive to this family (Figure 3A and Additional file 1: Table S4). However, comparing the insertion points in Beijing and non-Beijing strains, only Beijing presented the deletion of RD152 generated by reorganization of two IS*6110*. Nonetheless, locations within Rv1371, Rv2016, *ctpD*, *idsB* genes and between the two IS*1532* were only observed in Beijing strains (Figure 3A and Figure 3B, and Additional file 1: Table S4 and S5) as observed with the 8 Beijing isolates earlier in this work. Due to the high frequency of all of these IS*6110* locations, these regions seem to be specific hot-spots in Beijing strains. These results agree with Thorne *et al*. that showed the presence of some insertion points conserved within genetic lineages [41]. With the exception of the characteristic A1 point, to our knowledge, this is the first time that specific insertion points are described for Beijing strains, allowing us to speculate their possible relation with the fitness advantages of this family.

On the other hand unique locations were observed in *papA*4 and Rv2957 genes (N4 strain), interrupting *mez* and *PPE49* genes (CAM22 strain) and in the intergenic region of Rv1542c:Rv1543 (W4 strain) (Additional file 1: Table S5). We corroborated the uniqueness of these sites, after analyzing the literature [10,11,13,16-18,20,22-24,27-35], as was the case for the copy located upstream of Rv2180c gene in GC1237 [10] (Figure 1 and Figure 2). The data suggests that although IS*6110* has preferential genomic regions, its insertion is sufficiently random that could generate differences among strains of the same family.

### IS*6110* distribution in the eleven functional categories of *M*. *tuberculosis*

In the analyzed 61 strains, we observed that 58% of the IS*6110* insertions (94 of 160 points) ocurred in ORFs (Additional file 1: Table S4). The interruption of coding regions can be seen as a naturally occurring knock-out assay and could provide information on non-essential genes for mycobacteria capacity of infection in human host, as all the studied strains are clinical isolates. Given that the ORFs represent 91% of *M*. *tuberculosis* genome [9], our data suggest that transposition is relatively more frequent in intergenic than in intragenic regions. The higher number of intergenic events is likely due to selection, as they are less probable to be deleterious than those that occur within genes. Such events could increment the probability of insertion of IS*6110* in possible promoter regions, which could influence the expression of neighbouring genes.

Our results agree with other studies which found that 58% of discrete IS*6110* insertion sites occurred within coding regions in *M*. *tuberculosis*[32] and in *M*. *bovis* strains [22].

We observed that IS*6110* does not interrupt ORFs of 3 of the 11 functional categories: the stable RNAs, information pathways and IS and phages (http://tuberculist.epfl.ch/). Only one gene, Rv2103c, of the virulence category was disrupted (Additional file 1: Table S4). The distribution of the interrupted genes of the rest of the categories was markedly similar to the percentage of ORFs in *M*. *tuberculosis* genome with high frequency of disrupted genes in categories of hypothetical proteins and the intermediary metabolism and respiration. The details of the distribution in the 11 categories are indicated in Additional file 1: Table S4.

Several studies have indicated that it is frequent to find IS*6110* inserted in PPE/PE genes [17,21,22,27,31,35]. These genes are associated with antigenic variation in *M*. *tuberculosis*[42] but it has been suggested that the disruption of a member of this family would not be expected to produce severe disadvantages as it can be compensated by another member [27]. We observed that the interrupted PPE genes were PPE16, PPE34, PPE38, PPE40 and PPE49 in several points and in both orientations and there were no disrupted PE genes (Additional file 1: Table S4). PPE40 has been suggested as an essential gene [43], but one of the studied strains (clinical isolate) presents a copy in it.

### Analysis of DRs flanking IS*6110*

After analysing the flanking sequences of each copy of IS*6110* in the studied strains, we observed that 80% of the different insertion points (128 of 160) were flanked by DRs of 3–4 bp (Additional file 1: Table S4), indicating that these were the result of transposition events. The other 32 copies without detected DRs showed genomic reorganizations or loss of genomic regions. The deletions of RD152 and RD207 are examples of recombination between two adjacent copies of IS*6110*[44]. The IS*6110* without DRs localized between Rv0794c:Rv0797, is in the opposite orientation to the IS*6110* in H37Rv and in identical position as the copy in the reference Beijing GC1237 [10], and it is associated with genomic reorganization of this region. Due to the high number of copies of IS*6110* per strain, it was possible to observe copies flanked by DRs and other copies without DRs suggesting that the probability of rearrangement between copies rises when the number of those increases, producing more variability among strains. This finding is in agreement with different studies indicating that strains with a high number of IS*6110* copies have lost genomic regions more often than strains with only few copies [10,22]. Although in some of the studied HCS the number of the located IS*6110* copies was less than 5, some of them were observed without DRs, corroborating the idea that the probability of rearrangement processes between copies rises when the number increases. This is in agreement with other studies which localized IS*6110* in LCS and observed that all copies were flanked by DRs [22,45]. In one copy of IS*6110* we detected DRs of 5 nucleotides (Additional file 1: Table S4).

### Twenty percent of the located copies of IS*6110* in the studied strains could act as mobile promoter

As we reported previously in this work, IS*6110* is relatively more frequent in intergenic regions, increasing its probability of being inserted in promotor regions, influencing the expression of neighbouring genes. Different studies have indicated that when IS*6110* is inserted in the same orientation as, and close enough to, a downstream gene could potentially function as a promoter [10,23,24]. The orientation of the 440 located copies of IS*6110* in the 61 strains and the distance to the close genes were analysed in order to test the promoter function of this element. Thirty-two locations of IS*6110* were located close enough to (less than 400 bp) and in the same orientation as the neighboring gene (Additional file 1: Table S4). Of the 32 candidate locations to act as a mobile promoter, 4 of them were observed at a frequency of 3.2% and 23 at 1.6%. The remaining 5 locations were observed in a higher number of strains with 3 of these locations observed in non-Beijing strains and 2 in Beijing strains (Additional file 1: Table S4). Of note, one of the two frequent locations among Beijing strains corresponds to the copy located in *ctpD* gene and upstream Rv1468c gene and it has already been demonstrated that this copy is acting as a promoter inside monocytes [23]. The fact that this location is quite frequent in Beijing genotype and even is specific of this family, could be one of the special features of the Beijing family in terms of virulence and transmission.

Two of the 61 strains presented a copy of IS*6110* in the promoter region of *phoP* gene (Additional file 1: Table S4). The multidrug resistant (MDR) strain *M*. *bovis* B or MBZ responsible for large tuberculosis outbreaks in Spain has a copy of IS*6110* located 75 bp upstream the *phoP* gene [24]. Soto *et al*. demonstrated that this IS*6110* causes an increment in the transcription of *phoP*[24], which could have an important consequence as the product of this gene is an important transcriptional regulator [46]. One of the two strains (non-Beijing strain HMS 2405) presents a copy of IS*6110* 196 bp upstream and in the same orientation as *phoP* gene. In this case, the *phoP* promoter region is not interrupted, as IS*6110* is inserted upstream of transcription starting points, tsp1 and tsp2, of this gene and could also provide an additional tsp. Although this point is different from that in MBZ, HMS 2405 could be an interesting candidate for studying the effect of IS*6110* in *phoP* gene in a drug-susceptible *M*. *tuberculosis* strain.

### Generation of an accurate dendrogram based on IS*6110* points of insertion

**Figure 4:**
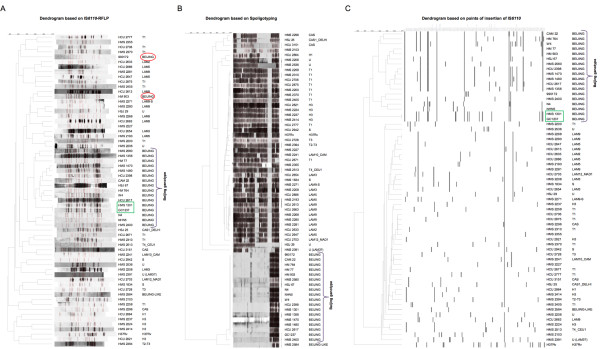
**Comparison of three dendrograms based on IS*6110*-RFLP (A), spoligotyping (B) and points of insertion of IS*6110 *(C) of the 61 strains used in this work.** The IS*6110*-RFLP, spoligotyping and points of insertion of IS*6110* of GC1237 and H37Rv strains were included in the dendrograms. The Beijing genotype is indicated in the three cases.

After observing that the 17 studied Beijing strains shared a high number of locations not present in non-Beijing strains, we decided to group the 61 clinical isolates based on their insertion points. The obtained dendrogram, using Bionumerics program, (Figure 4A) shows that Beijing strains were grouped better than when using the IS*6110*-RFLP classification system (Figure 4B). Furthermore, some non-Beijing strains were also grouped in families despite having very few located copies of IS*6110*. This fact could indicate that probably each family has their preferential sites of insertion. However, the grouping based on IS*6110* locations in non-Beijing families was not perfect due to the lack of information on all their points of insertion.

The classification technique based on IS*6110* insertion points developed in this study allows grouping the strains in families as spoligotyping and provides information on the clone or strain as IS*6110*-RFLP. As Figure 4A shows, although IS*6110*-RFLP groups genotypes, if the IS*6110*-RFLP of a Beijing strain is quite different from other Beijing strains, the strain is classified as distant from the group. Based on the results obtained in this study, the development of a 96-well plate with pairs of primers which amplify copies of IS*6110* characteristics of each family could allow to obtain a dendrogram in one day. This PCR-based method is quick, accurate and economic and possible to complete in one day.

## Conclusions

This study provides a detailed analysis of the locations of IS*6110* in *M*. *tuberculosis* Beijing genotype compared to other HCS, including the exact insertion sites, the flanking DRs, the orientation and the distance of IS*6110* to neighbouring genes. In general, the insertion of IS*6110* is random generating inter- and intrafamily strain differences. We have obtained a dendrogram based on insertion points which differentiates the Beijing genotype from the rest of the families. We describe for the first time specific insertion sites in Beijing genotype. The detection of unique points for concrete clinical isolates can be used as a useful tool in the rapid diagnosis allowing the identification and differentiation of a particular strain.

## Methods

### Bacterial selected strains, culture media, growth conditions and isolation of mycobacteria genomic DNA

Sixty-one *M*. *tuberculosis* clinical isolates were used in this work. The 61 isolates comprised 17 Beijing and 44 non-Beijing strains. Among the 17 Beijing strains 8 (NHN5, HM77, HM903, HM764, 990172, W4, N4 and CAM22) from Europe were previously selected as representative of this genotype using several typing methods. These 8 strains were selected as they share 80% or more of identity with Beijing strains from Shanghai area, China. The rest of the strains, each representing a different cluster, were selected for their high number copies of IS*6110* (ten or more) and were collected from Hospital Universitario Miguel Servet (HMS), Hospital Clínico Universitario Lozano-Blesa (HCU) from Zaragoza and Hospital General San Jorge from Huesca (Spain). Finally, *M*. *tuberculosis* H37Rv and *M*. *tuberculosis* GC1237 were used as control strains. An internal control, HMS 1301, was included in the study as its IS*6110*-RFLP is identical to GC1237 control strain. Mycobacterial strains were grown at 37°C in Middlebrook 7H9 broth supplemented with ADC and 0.05% Tween 80. More information of the clinical isolates used in this work is included in Additional file 1: Table S1.

### Isolation of genomic DNA

Genomic DNA of mycobacterial strains was isolated using the CTAB method as previously described by van Soolingen *et al*. [47].

### Localization of the copies of IS*6110* insertion sequence in the eight Beijing strains selected as representative strains of this genotype

The first step of this work was to localize copies of IS*6110* in the 8 representative Beijing strains by two methods, both based on PCR. A first research was conducted by Ligation-mediated PCR (LMPCR) as previously described by Prod’hom *et al*. [48]. Briefly, genomic DNA was digested with *Sal*I enzyme and the digestions were then subjected to PCR with ISA1 or ISA3 specific primers for IS*6110* directed outwards [45] and the common linker primer SALGD (Additional file 1: Table S2). PCR products were purified using GFX PCR DNA gel band purification kit (Amersham Pharmacia Biotech) and the restriction enzyme ExoSAP-IT® (Affymetrix). The amplified products were sequenced with the corresponding oligonucleotides and when a match was found, additional primers (Additional file 1: Table S2) were designed and used with the 8 strains to verify whether the point of insertion was present in other of the 8 strains. These primers amplify the completed sequence of IS*6110* and approximately 300 bp of both flanking sequences (Additional file 1: Table S2).

Secondly, PCRs were performed with specific primers (Additional file 1: Table S2), designed in a previous study for amplifying the locations of IS*6110* in 210, W [31] and GC1237 strains [10]. PCRs were carried out in a total volume of 25 μl, containing 50 ng of DNA, 2.5 μl of 10x PCR buffer, 200 μM dNTPs, 12.5 pmol of each primer and 1 U Taq Gold polymerase (Roche). Before the amplification, the template was initially denatured by incubation at 94°C for 9 min then the amplification was performed for 35 cycles of 94°C for 30 s, corresponding annealing temperature for 30 s, and 72°C for 1 to 2 min depending on the amplified product. After the last cycle, the samples were incubated at 72°C for 10 min.

The genomes of H37Rv and GC1237 were used as control in both cases.

### Sequence analysis of points of insertion of IS*6110* in the DNA database and in the available literature

The points of insertion of IS*6110* of the reference sequenced strains: Beijing (210, 02_1987, 94_M4241A, HN878, R1207, T85, X-122, W and W-148 ) and non-Beijing (98-R604 INH-RIF-EM, BTB05-552, BTB05-559, C, CDC1551, CDC1551A, CPHL_A, EAS054, F11, GM 1503, K85, KZN 605, KZN R506, KZN V2475, KZN 1435, KZN 4207, NCGM 2209, str.Haarlem, S96-129, SUMu001, SUMu002, SUMu003, SUMu004, SUMu005, SUMu006, SUMu007, SUMu008, SUMu009, SUMu010, SUMu011, SUMu012, T17, T46 and T92) were obtained comparing the flanking regions of each IS*6110* in the genome sequences with the reference strain H37Rv using NCBI genetic sequence database (GeneBank) (http://www.ncbi.nlm.nih.gov/genome/166).

Moreover, available literature was analyzed to identify any further point of insertion of this sequence not described in the DNA database [10,11,13,16-18,22-24,27-35].

After this sequence analysis, when a point of insertion of IS*6110* of a Beijing strain (of GeneBank or available literature) was found outside of the amplified regions of our primer collection, additional primers were designed and included in the collection (Additional file 1: Table S2). In addition, flanking primers of DK regions [28] were designed to detect whether these frequent locations of IS*6110* in LCS were also present in the HCS selected in this study. Moreover, the primers designed for the study of locations of IS*6110* in clinical isolates of *M*. *bovis* with human host [22] were also included to study whether preferred locations of IS*6110* in these *M*. *bovis* strains were also present in the selected *M*. *tuberculosis* strains.

### Localization and analysis of copies of IS*6110* in the sixty-one selected clinical isolates

The localization of copies of IS*6110* in the 61 clinical isolates was carried out by PCR as previously describe in this work with all the pairs of oligonucleotides of the generated primer collection (Additional file 1: Table S2). H37Rv and GC1237 were used as external controls and HMS 1301 as internal control as this strain presents the same IS*6110*-RFLP as GC1237. The 8 representative Beijing strains studied before in this work by LMPCR and specific PCR were again included in this part of the study. The PCR products which might include an IS*6110* were sequenced with IS61 and IS62 primers (Additional file 1: Table S2).

### Determination of direct repeats (DR) and analysis of the flanking regions of each copy of IS*6110* in the genomes

The DRs generated by the mechanism of the transposition of IS*6110* were determined with the sequence analysis of the flanking regions of each copy of IS*6110* in the genomes.

### Dendrogram based on the points of insertion of IS*6110*

The informatic analysis of spoligotyping results is carried out by Bionumerics program and is based in presence/absence or numerical analysis 1/0 of a specific sequence of *M*. *tuberculosis*. Based on this idea, all the obtained IS-locations were arranged in columns on an excel sheet and the 61 strains (also the two controls GC1237 and H37Rv) were arranged in rows. When a strain presented a point of insertion, it was given the number 1 and if not, it was assigned the number 0. After that, the data was introduced in SpolDB4 database as a new informatic event, included to the IS*6110*-RFLP and the spoligotyping of each strain and analyzed by Bionumerics program.

## Competing interests

The authors declare that they have no competing interests.

## Authors’ contributions

Conceived and designed the experiments: HA, IO. Performed the experiments HA. Analyzed the data HA, SS, CM, IO. Wrote the paper: HA, SS, CM, IO. All authors read and approved the final manuscript.

## Supplementary Material

Additional file 1 Supplementary informations. Click here for file
